# The System Profile of Renal Drug Transporters in Tubulointerstitial Fibrosis Model and Consequent Effect on Pharmacokinetics

**DOI:** 10.3390/molecules27030704

**Published:** 2022-01-21

**Authors:** Birui Shi, Yan Zhang, Baolin Huang, Huiping Lin, Qiong Zhou, Yujue Wang, Zheng Cai, Menghua Liu

**Affiliations:** 1 Biopharmaceutics, NMPA Key Laboratory for Research and Evaluation of Drug Metabolism, School of Pharmaceutical Sciences, Southern Medical University, Guangzhou 510515, China; stone0907mua@163.com (B.S.); smuzy249210661@163.com (Y.Z.); hbl_2307@163.com (B.H.); lhp_645572@163.com (H.L.); 2 Integrated Traditional Chinese and Western Medicine Hospital of Southern Medical University, Southern Medical University, Guangzhou 510515, China; zqiong1976@126.com; 3 Laboratory Animal Management Center, Southern Medical University, Guangzhou 510515, China; wangyujue@smu.edu.cn

**Keywords:** drug transporters, pharmacokinetics, OCT2, MATE1, P-gp

## Abstract

With the widespread clinical use of drug combinations, the incidence of drug–drug interactions (DDI) has significantly increased, accompanied by a variety of adverse reactions. Drug transporters play an important role in the development of DDI by affecting the elimination process of drugs in vivo, especially in the pathological state. Tubulointerstitial fibrosis (TIF) is an inevitable pathway in the progression of chronic kidney disease (CKD) to end-stage renal disease. Here, the dynamic expression changes of eleven drug transporters in TIF kidney have been systematically investigated. Among them, the mRNA expressions of Oat1, Oat2, Oct1, Oct2, Oatp4C1 and Mate1 were down-regulated, while Oat3, Mrp2, Mrp4, Mdr1-α, Bcrp were up-regulated. Pearson correlation analysis was used to analyze the correlation between transporters and Creatinine (Cr), OCT2 and MATE1 showed a strong negative correlation with Cr. In contrast, Mdr1-α exhibited a strong positive correlation with Cr. In addition, the pharmacokinetics of cimetidine, ganciclovir, and digoxin, which were the classical substrates for OCT2, MATE1 and P-glycoprotein (P-gp), respectively, have been studied. These results reveal that changes in serum creatinine can indicate changes in drug transporters in the kidney, and thus affect the pharmacokinetics of its substrates, providing useful information for clinical use.

## 1. Introduction

Drug combination is a joint therapeutic scheme for the treatment of clinical diseases. However, the incidence of drug–drug interactions (DDIs) is remarkably increasing, resulting in a variety of adverse reactions, even threatening human life [1]. Drug transporters are one of the main targets for DDIs. Kidney tissue, the main excretory organ in the body, shows the distribution of drug transporters. Many drugs (including organic anion drugs, organic cationic drugs, and peptide drugs) are mediated by drug transporters concentrated in proximal renal tubules during renal excretion [2]. Once the expression of drug transporters changes, it binds to affect the pharmacokinetics of drugs. Therefore, the Food and Drug Administration and National Medical Products Administration of China have pointed out that eleven drug transporters in the kidneys, including organic anion transporter 1 (OAT1), organic anion transporter 1 (OAT3), organic anion transporter polypeptide 4C1 (OATP4C1), organic cation transporter (OCT2), P-glycoprotein (P-gp), breast cancer resistance protein (BCRP), multi-drug and toxin extrusion protein 1 (MATE1), multi-drug and toxin extrusion protein 2-K (MATE2-K), organic anion transporter 4 (OAT4), multidrug resistance-associated protein 2 (MRP2) and multidrug resistance-associated protein 4 (MRP4), need to be researched for drug applications [3,4].

CKD is widespread in the world, affecting nearly 13% of the population, and CKD has become a global public health problem [5]. According to the online data of the Centers for Disease Control and Prevention, the number of CKD deaths increased by about 12% from 2011 to 2018, ranking ninth in the top ten fatal diseases [6]. Tubulointerstitial fibrosis (TIF) is a common pathological change in CKD progression to end-stage renal disease [7,8]. With kidney damage, CKD is often accompanied by hypertension, cardiovascular disease, diabetes, and other complications. Therefore, combination therapy is a frequent method for patients with CKD [9,10]. In the clinic, the drug administration in patients with CKD is very cautious. Creatinine (Cr) is an endogenous substance that was filtered out through the glomerular [11]. Creatinine clearance (Ccr) is commonly used to evaluate renal function [12,13]. When the drug is eliminated, primarily by glomerular filtration, the clinical administration schedule could be adjusted according to the patient’s Cr/Ccr under pathological conditions. However, there have been no clear reports on the changes in drug transporters excreted by drug transporters in vivo, the changes in drug transporters expression in TIF, and the relationship between Cr/Ccr and drug transporters. Glomerular filtration rate (GFR) and proteinuria are still widely used diagnostic indicators, but these two indicators occur late in the disease. Therefore, it is urgently needed to explore the relationship between new indicators and transporters.

Therefore, this study focuses on the relationship between kidney transporters and Cr/Ccr in unilateral urethral obstruction animal model, to provide useful data for the use of clinical drugs and drug combination.

## 2. Results

### 2.1. The Renal Parameters in TIF Rats

**Figure 1:**
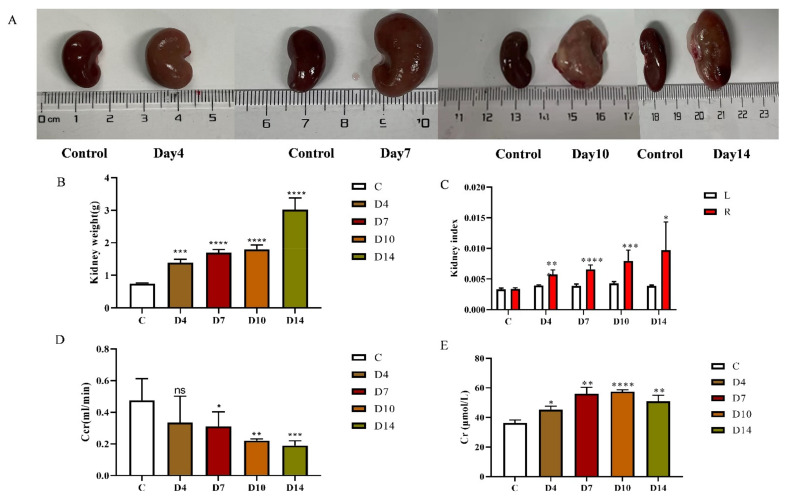
The renal parameters for TIF model. (**A**)The right kidney of TIF group compared with that of the control group on 4th, 7th, 10th, and 14th days. C: Control; D4: Day 4 of TIF model; D7: Day 7 of TIF model; D10: Day 10 of TIF model; D14: Day 14 of TIF model. (**B**) In the anatomical model group on different days, the ligation kidney of rats was weighed, and the weight obtained was compared with that of the control group. (**C**) Renal index was calculated as the ratio of the weight of the left kidney and the ligation kidney to body weight. Data were expressed as mean ± SD. L: left kidney, R: ligated kidney. (**D**) Changes in Cr in serum concentration of rats at different modeling time, compared with the control group. (**E**) Changes in Ccr in serum concentration of rats at different modeling times, compared with the control group. **** *p* < 0.0001 *** *p* < 0.001, ** *p* < 0.01, * *p* < 0.05, ^ns^ *p* > 0.05.

To observe the dynamics of kidney tissue in TIF rats, the orbital blood and kidneys were harvested on the 4th, 7th, 10th, and 14th days after modeling in the model group. The renal structure was illustrated in Figure 1A. With the increase in modeling time, the right kidney of rats showed an obvious swelling, translucent epidermis, light color, cystic, containing brown turbid liquid. With the increase in modeling time, compared with the control group, the wet weight of the right kidney in the model group increased by approximately 1.51–3.05-fold, and reached the maximum value on the 14th day (Figure 1B). With the increase in modeling time, the coefficient of right kidney of rats increased by about 1.017–2.507-fold compared with that of the left kidney (Figure 1C). The measurement of Cr in serum revealed that Cr concentration increased 1.24–1.58 times with the increase in modeling time (Figure 1D). On the contrary, with the increase in modeling time, Ccr decreased to 39.8–70.7% (Figure 1E).

### 2.2. Histopathological Findings

**Figure 2:**
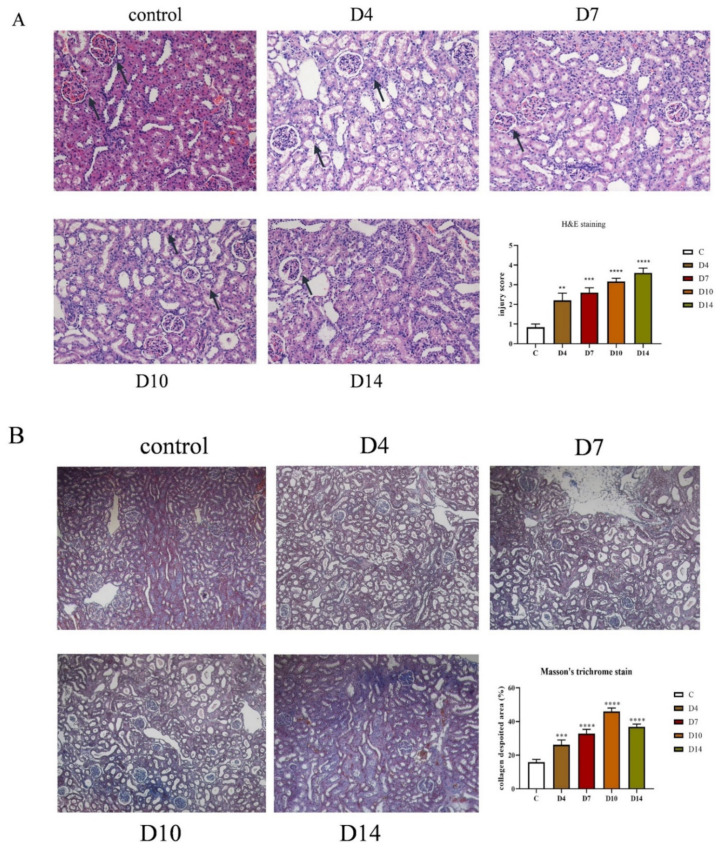
Histopathological results. Sections of the right kidney of on rats at 4th, 7th, 10th, and 14th days were taken to H&E staining (**A**). Scale: 600 μm (200×). Histopathological changes in kidney sections were scored as a semi-quantitative percentage of damaged area: 0, normal; 1, cortical area <25%; 2, cortical area 25–50%; 3, the cortical area is 50–75%; 4, cortical area >75%, compared with control group. (**B**) Sections of the right kidney of on rats at 4th, 7th, 10th and 14th days were taken for Masson staining. Fibrosis area was quantified by Image J Pro Plus 6.0 compared with the control group. **** *p* < 0.0001 *** *p* < 0.001, ** *p* < 0.01.

The H&E staining and Masson staining were employed to examine the pathological morphology of the tissues as well as fibrocyte collagen precipitation, respectively, as shown in Figure 2A. The H&E results showed that the kidneys in TIF rats exhibited glomerular fibrosis with cystic changes, glomerular enlargement with massive inflammatory cell infiltration, and widening of the renal interstitial space, with the increase in modeling time. They were then scored for pathological damage (Figure 2A), which showed that both kidney injuries increased as modeling time increased. Masson’s results indicated that renal tubular dilatation, widening of the renal interstitial space, and an obvious increase in collagen fibers in the renal interstitium were seen in the obstructed side kidneys of the model group compared with the control group (Figure 2B). As the modeling time increased, the fibrosis area increased by 12%, 19%, 35%, and 38% (Figure 2B).

### 2.3. The Variation of mRNA Expression in the TIF Rats

**Figure 3:**
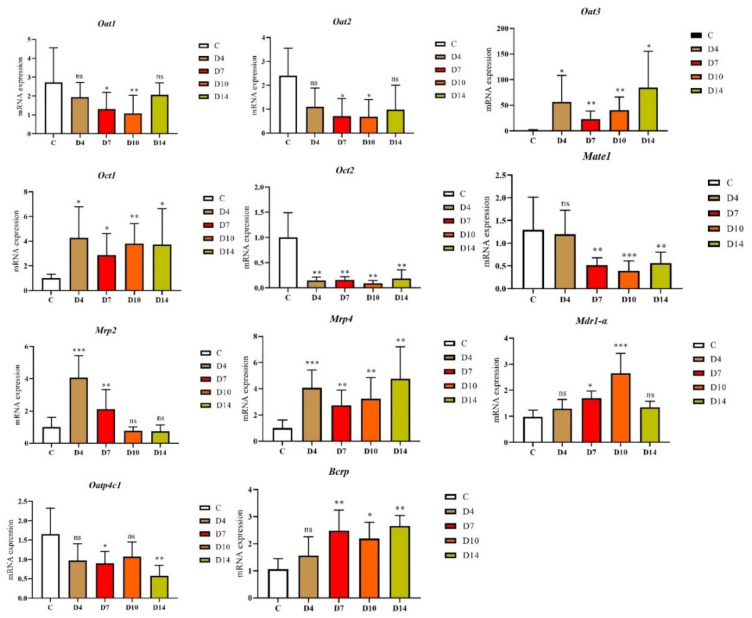
The variation in mRNA expression of drug transporter in TIF kidney. Samples were detected on 4th, 7th, 10th and 14th days. C: Control; D4: Day 4 of TIF model; D7: Day 7 of TIF model; D10: Day 10 of TIF model; D14: Day 14 of TIF model. *** *p* < 0.001, ** *p* < 0.01, * *p* < 0.05, ^ns^ *p* > 0.05.

The mRNA expression of 11 drug transporters (*Oat1, Oat2, Oat3, Oct1, Oct2, Oatp4C1, Mate1, Mrp2, Mrp4, Mdr1-α and Bcrp*) in TIF kidney was determined by RT-qPCR. As showed in Figure 3, *Oat1, Oat2, Oct1, Oct2, Oatp4C1* and *Mate1* mRNA expressions were downregulated, while *Oat3, Mrp2, Mrp4, Mdr1-α* and *Bcrp* expressions were upregulated. Among them, *Oat1* (39.6–76%) and *Oat2* (28–46%) indicated a gradual decrease, first with the increase in modeling time, and then a slight callback on the 14th day, where *Oat3* (1639–5985%) and *Mrp2* (75.32–408%) showed a significant increase and then a slow trend, while the mRNA expression of *Oct2* (9–18%) and *Mate1* (30.1–92.3%) was significantly reduced. The mRNA expression of *Oatp4C1* significantly increased on the 4th day and began to decline on the 7th day, and there were no significant differences between *Oatp4C1* and the control group on the 10th and 14th days. *Mrp4* increased sharply in model groups.

### 2.4. The Correlation of Renal Transporters and Cr/Ccr in the Pathological State of Renal Fibers

**Figure 4:**
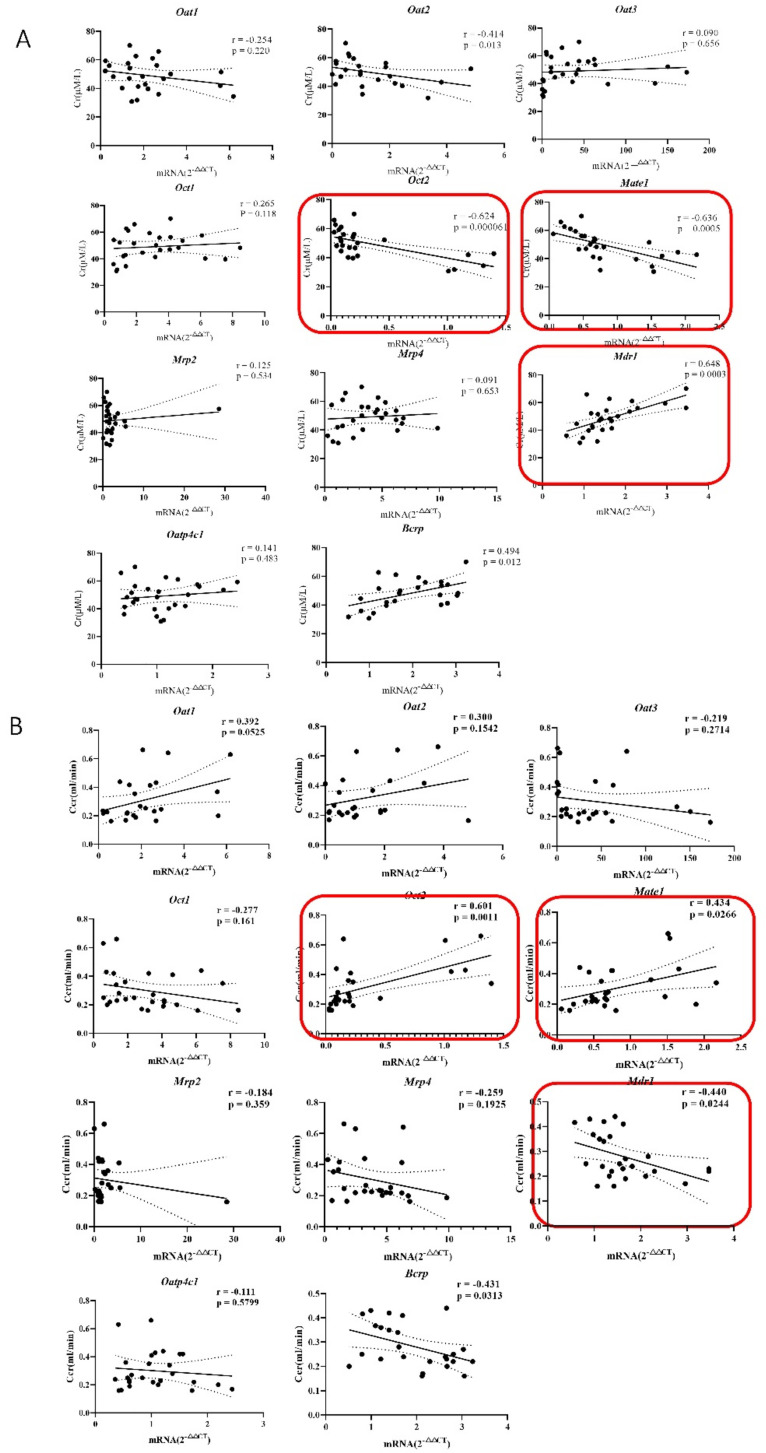
(**A**) Real-time q-PCR analysis showed that the mRNA content of these transporters was 2^−∆∆Ct^ relative to the mRNA β-actin expression, and Pearson correlation was used to analyze the dynamic changes between the main kidney transporters and Cr. A correlation analysis of the relative size of 2^−∆∆Ct^ between the changed transporter and β-actin and Ccr was conducted. (**B**) The mRNA expression of transporter was detected on the 4th, 7th, 10th, and 14th days, and then the correlation between the expression value of transporter and the Ccr rate was analyzed.

Pearson correlation analysis was utilized to explore the relationship between renal transporter variation and Cr/Ccr under pathological conditions. The analysis result explained that *Oct2* and *Mate1* were highly negatively correlated with Cr (Pearson coefficient r >0.6, *p* ≤ 0.05), Among them, *Oct2* (r = 0.624, *p* = 0.000061), *Mate1* (r = 0.636, *p* = 0.0005), *Oat2* (r = 0.414, *p* = 0.013) were moderately related, and the rest were all less than 0.3. *Mdr1-α* was positively correlated with correlation coefficients lower than 0.5 (Figure 4A), such as *Bcrp* (r = 0.49, *p* = 0.012), which showed a medium relationship, but all others were less than 0.3 without a significant difference. The correlation between Ccr and transporters further confirmed these results. *Oct2* (r = 0.601, *p* = 0.0011) *Mate1* (r = 0.434, *p* = 0.0266), *Mdr1-α* (r = 0.440, *p* = 0.0244) (Figure 4B). In brief, the above results showed that renal transporters were related to Cr and Ccr, and *Oct2*, *Mate1* and *Mdr1-α* were strongly correlated.

### 2.5. The Correlation of Renal Transporters, Cr and Renal Fibers in the Pathological State of Renal Fibers

**Figure 5:**
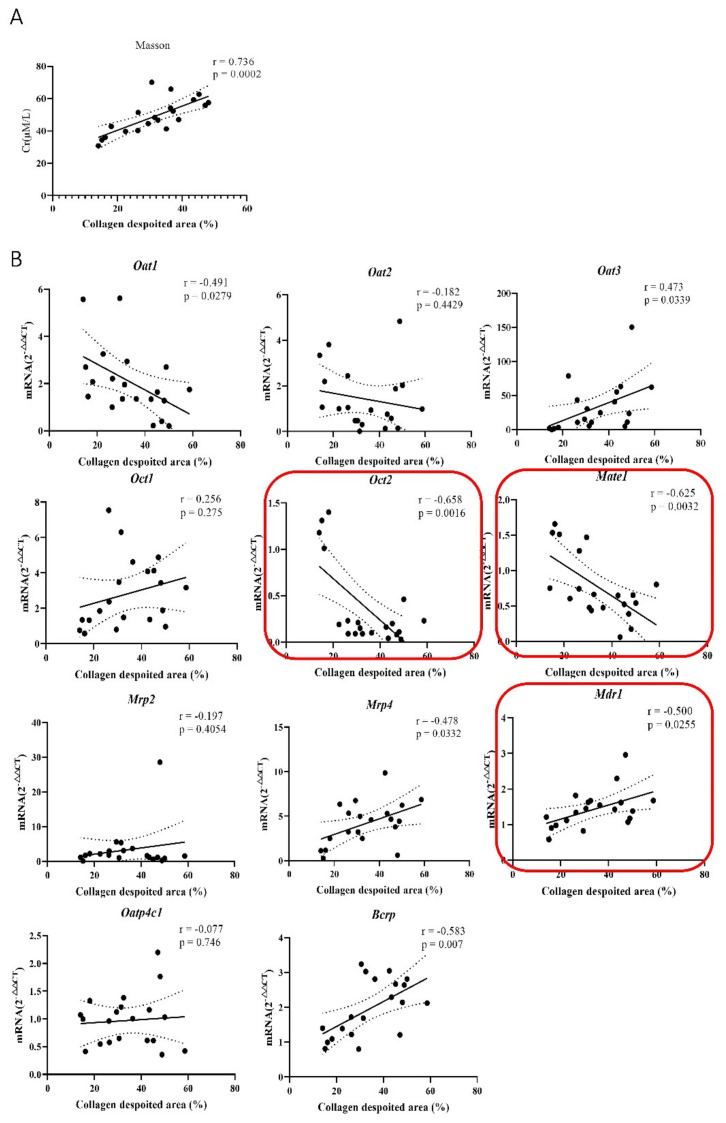
(**A**) The expression of 11 transporters in different groups was correlated with the results of Masson staining. (r > 0.6, strong correlation, 0.4 < r < 0.6, strong correlation, 0.2 < r, weak correlation) (**B**) Correlation between renal fibrosis and dynamic changes in Cr.

Further, we conducted a correlation analysis of the dynamic change in Cr and the degree of fibrosis, and the results exhibited that the degree of fibrosis was significantly positively correlated with the dynamic change of Cr (r = 0.736, *p* ≤ 0.05) (Figure 5A). We also detected a relationship between transporters and the degree of renal fibrosis. This showed that *Oct2* (r = 0.751, *p* = 0.0001), *Mate1* (r = 0.744, *p* = 0.0002), *Mdr1-α* (r = 0.597, *p* = 0.0055) were highly correlated with fibrosis, which were consistent with that of Cr/Ccr (Figure 5B).

### 2.6. PK of Renal OCT2, MATE1, P-gp Substrates in the TIF Rats

**Figure 6:**
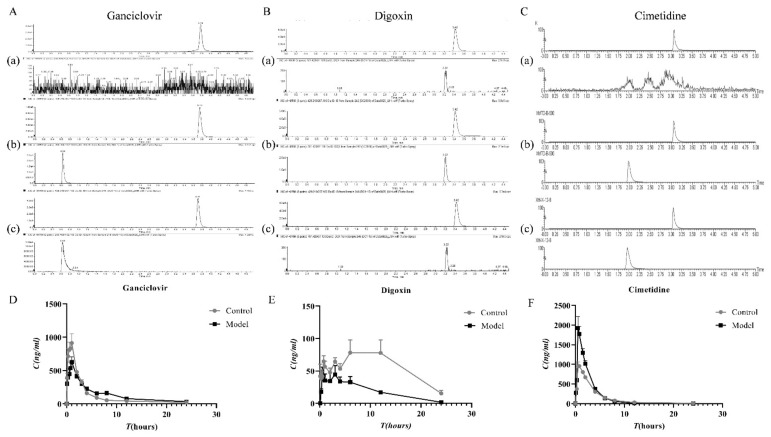
PK changes of renal OCT2, MATE1, P-gp substrates in the TIF model. (**A**)/(a) blank plasma + ebesartan (**A**)/(b) Blank plasma + Ganciclovir standard + internal standard (**A**)/(c), plasma sample (**B**)/(a) blank plasma + ebesartan (**B**)/(b) Blank plasma + Digoxin standard + internal standard (**B**)/(c), plasma sample (**C**)/(a) blank plasma + ebesartan (**C**)/(b) Blank plasma + Cimetidine standard + internal standard (**C**)/(c), plasma sample (**D**) the drug-concentration time curve of ganciclovir. (**E**) the drug concentration-time curve of Digoxin. (**F**) the drug-concentration time curve of cimetidine.

*Oct2*, *Mate1* and *Mdr1-α* regulate the expression of OCT2, MATE1 and P-gp proteins in vivo. To determine the influence of changes in transporters on pharmacokinetic parameters under pathological conditions, three typical substrates for OCT2, MATE1 and P-gp were selected for pharmacokinetic studies. A methodological verification of the three drugs was conducted (Table 1, Table S1, Figure 6A–C) and the detection method met the methodological requirements.

The results exhibited that the *AUC* of cimetidine (substrate of OCT2) in the model group increased 1.49 times compared with the control group (Figure 6D). The value of renal clearance (*Cl_r_*) in the model group decreased by 20.5%, which may be linked to the decreased expression of OCT2 protein in the kidney. Digoxin was a typical substrate of P-gp. Its *AUC* value reduced by 3.138-fold, while *Cl_r_* value increased by 2.6-fold, which might be related to the increased expression of P-gp in TIF rats. Ganciclovir is a substrate of MATE1. The *AUC* of ganciclovir decreased by 11.3%, while *Cl_r_* did not significantly change. The pharmacokinetics parameters did not significantly change when ganciclovir was combined with some MATE1 inhibitors or substrates, which may be related to other excretory pathways in vivo.

## 3. Discussion

The extensive literature suggests that the expression of kidney transporters in a pathological state will change, for example, under the rat liver ischemia–reperfusion model [14]. This will lead to the up-regulation of MRP and the down-regulation of OCT2, while, for hyperuricemia rats, in acute kidney injury, P-gp, MRP2 and other transporters will be significantly upregulated [15,16]. These changes may be due to the activation or induction of some upstream nuclear receptors under pathological conditions, such as PPAR-α and other nuclear receptors and transcription factors, thereby regulating the expression of downstream transporters [17,18], LXR and FXR are associated with Abcg1 gene and *Abc*-related protein expression, and its expression can cause changes in downstream transporters. PXR is associated with *Slc*-related protein expression and *Abc*-related protein expression, just like Mdr-1α and *Slc16a1* [19,20].

Therefore, changes in the body or under certain inflammatory or pathological conditions may cause changes in the expression of some nuclear receptors in the pathway, thus leading to changes in the expression of other transporters [21]. In addition to the nuclear receptors referred to above, some inflammatory factors can also directly affect the expression of transporters. For example, TNF-α can inhibit the transcription of the tubule bile acid transporter *Abcb11*, bilirubin outlet *Abcc2*, and sterol transporter *Abcg5/8* in intestinal inflammation, cholestasis, or the activation of hepatic macrophages, and thus affect the expression of transporters [22]. Therefore, the present study constructed a classical renal interstitial fibrosis model to explore the changes in renal transporter expression in rats under the TIF model.

Since Cr and Ccr are commonly used indicators to evaluate renal function, this experiment wanted to explore the change rule of Cr and Ccr and the expression of various transporters under the renal interstitial fibrosis model, and whether the expression changes in major transporters in kidney could be inferred through the detection of Cr and Ccr. Therefore, in this paper, the dynamic changes in transport proteins under the TIF model were related to Cr and Ccr by correlation analysis, and transport proteins were found that were highly correlated with Ccr. Transporter inhibitors are compounds that competitively bind or inhibit transporter activity [23,24]. Therefore, in the case of multi-disease combination, there will be interactions between drugs, such as P-gp [25,26], which has a variety of inducers in vivo, including antibacterial drug rifampicin, anti-tumor drug vincristine, doxorubicin, cardiovascular drug verapamil [27], hyperlipidemia drug atorvastatin [28], etc., which can induce the overexpression of P-gp in vivo. As a result, the pharmacokinetics parameters of drugs such as digoxin in vivo are significantly changed, while digoxin has a narrow treatment window, and the blood concentration of digoxin will be greatly reduced in a multi-drug combination, so that digoxin cannot play a therapeutic role. In many studies, the combination of naproxen and other agents with a typical OCT2 substrate (cimetidine) increased the plasma concentration of cimetidine, thereby separately increasing the toxicity of cimetidine [29]. Ganciclovir [30], its pharmacokinetic behavior in some studies [31,32], and part of its MATE1 inhibitors or substrate share, showed no significant change in pharmacokinetics parameters, which may be related to other excretory pathways in the body [33]. In addition, this paper also examines the ligation of the bilateral renal compensatory, where the transporter will affect the elimination of the substrate, and the results showed that the left kidney transporter expression showed no obvious change. We also considered the effect of absorption of drug excretion, in view of the selected several drugs in the clinic, which are mainly for oral use, and choosing the means of lavage for pharmacokinetics validation. In addition, the specific transporters OCT2, Mate1 and MDR1-α showed a high correlation with renal fibrosis. Therefore, we can deduce the renal fibrosis process from indicators such as blood creatinine/creatinine clearance. Further research will continue to focus on this aspect and deeply explore the mechanism of renal transporter expression changes under pathological conditions.

The study has several advantages and limitations. The advantages include the simplicity pf Cr in serum and Ccr, which can introduce a change in the transporter, as well as the transporter excretion of drug medication guides, without the need for a kidney biopsy. The first limitation is the change in renal fiber and Ccr constant transporter. It is unknown whether his drugs change, as their pharmacokinetics parameters were not studied. Second, the study used TIF rats in the 14th-day group, without considering the changes in pharmacokinetics parameters in other groups. In this study, only rats were used for transport experience, so this was not verified in clinical patients. Therefore, the results of this study may not be applicable to the whole population. As the next step, we will continue to supplement pharmacokinetics experiments to study whether the pharmacokinetics of substrates of several other transporters with a low correlation with Ccr will change, and verify this using in vitro experiments.

## 4. Materials and Methods

### 4.1. Chemicals and Regents

Chloral hydrate (≥99% in purity) was provided by Guangzhou Youbang Biotechnology Co., Ltd. (Guangzhou, China). Cimetidine (≥99% in purity) and irbesartan (internal standard, >98% in purity) ware purchased from Shenzhen upno Biomedical Technology Co., Ltd. (Shenzhen, China). The kit for analysis of blood urea nitrogen (BUN) and Cr was purchased from Nanjing Jiancheng Bioengineering Institute (Nanjing, China). The animal total RNA isolation kit was provided by Foregene Co., Ltd. (Chengdu, China). All other chemical reagents were of chromatographic or analytical grade and were commercially available.

### 4.2. Animals

Healthy male Sprague-Dawley rats (SD rats, aged 7–8 weeks, weight 180–220 g, certification: SCXK-Yue-2016-0041) in specific pathogen-free grade were available from the Experimental Animal Center of Southern Medical University (Guangzhou, China). All experiments followed the National Institutes of Health Guidelines for the Care and Use of Laboratory Animals. All animals were housed in an air-conditioned room with the temperature at 23 ± 2 °C and a relative humidity of 40 ± 5%, under an alternating 12 h dark/light cycle. Animals had free access to food and water throughout the experiment.

### 4.3. Animal Experiment

Thirty SD rats were randomly divided into five groups (*n* = 6): control and TIF model groups analyzed on the 4th, 7th, 10th and 14th days. The rats in the model group were intraperitoneally anesthetized with 10% chloral hydrate at a dose of 0.3 mL/100 g [34]. Surgery was carried out as previously described. In the model group, the right ureter was exposed and ligated at two points with a 5–0 sterile suture along the lower pole of the right kidney. Then, the ureter was cut to prevent retrograde infection [35]. Two hours later, they were intraperitoneally injected with penicillin (1.6 million units dissolved in 8 mL of normal saline) for two consecutive days. Each rat was subcutaneously injected with 0.30 mL. On the 4th, 7th, 10th and 14th days after operation, blood was taken from the orbit of TIF rats. Urine was taken from the TIF rats placed in the metabolic cage for 24h. Heart, liver, spleen, lung, kidney, intestine and other tissues were dissected. The kidney tissues were weighed and the ratio of kidney weight to body weight was estimated. The right kidney was longitudinally reduced and fixed with paraformaldehyde. The rest was used only for a real-time quantitative polymerase chain reaction (RT-qPCR).

### 4.4. Histology Analysis

The kidney tissue was longitudinally cut, rinsed several times with cold PBS, and fixed overnight with 4% paraformaldehyde. Then, five pieces were cut out after paraffin embedding of the μM section. The renal tissue was stained with H & E at low power (10 × 10) The observation site was determined under a high-power microscope (10 × 20 and 10 × 40) and the target field of vision was selected to take 1–2 pictures. In H & E staining, the degree of renal injury was determined according to the size of glomeruli and the changes in renal tubules [36].

Masson staining: Image Pro Plus 6.0 software was used for quantitative analysis. The degree of renal interstitial fibrosis was evaluated based on the amount of collagen deposition (the percentage of the blue area in the whole cortex). Five different cortical fields were randomly selected from each slice (magnification 200 times). The area of fibrotic lesions was expressed as the percentage of fibrotic area in the whole cortex [37,38].

### 4.5. Detection of mRNA Expression: RT-qPCR

A total of 10-20 mg renal tissue samples were collected into the homogenization tube. Total RNA was extracted according to the protocol of animal tissue total RNA Extraction Kit (Foregene, Chengdu, China). A total of 1000 ng of total RNA was reverse-transcribed into cDNA using Evo m-mlv reverse transcription reagent (Accurate, Shenzhen, China). All subsequent RT-qPCR reactions were performed using 2 × Accurattaq Master Mix (Accurate, China), primer (designed and synthesized by Guangzhou Branch of Beijing Qingke Biotechnology Co., Ltd., Guangzhou, China, Table S2), ddH2O without ribonuclease, reaction volume 20 μL. The PCR was conducted on a rapid real-time PCR system (7500, Thermo Fisher Science, Waltham, MA, USA). At 50 °C The results were analysed under the conditions of C reaction for 3 min, 95 °C reaction for 3 min, 95 °C reaction for 10 s, and 60 °C reaction for 30 sec. The threshold period (CT) was recorded with 7500 fast system software version 2.3, and the multiple changes in mRNA expression were calculated according to the comparative CT method.

### 4.6. Pharmacokinetic Analysis

Thirty rats were split into six groups (*n* = 5). The rat model of TIF was established by unilateral ureteral obstruction surgical operation under sterile conditions according to previous research. On the 14th day after establishment of the model group, cimetidine (18 mg/kg), ganciclovir (45 mg/kg), and digoxin (5 mg/kg) were given orally in each group, respectively [39]. Blood samples were collected from the retroorbital sinus at the 0 min, 5 min, 15 min, 30 min, 45 min, 90 min, 120 min, 240 min, 360 min, 480 min, 720 min and 1440 min timepoints, and centrifuged immediately after collection (5000 rpm, 8 min), The obtained plasma was stored at −80 °C before any pharmacokinetics analysis. The plasma concentration of cimetidine in SD rats was established by high-performance liquid chromatography tandem mass spectrometry (UPLC-MS/MS).

### 4.7. Detection Method

For HPLC, System: Waters Acquity UPLC H-Class (UFLC Nexera, SHIMADZU, Kyoto, Japan); Chromatographic column: X Bridge C18 column (2.5 μm, 2.1 mm × 75 mm); column temperature: 30 °C; sample chamber temperature: 4 °C; injection volume 1 μL was used for accomplishing the chromatographic separation with mobile phase consisting of A: 0.1% formic acid water B: methanol.

In a positive ion mode, an API 4000 triple quadrupole tandem mass spectrometer (SCIEX, Framingham, MA, USA) with an ESI (AB SCIEX, Framingham, MA, USA) source was used, and the acquisition and analysis of data were carried out with Analyst 1.6.2 software (Applied Biosystems, Foster City, CA, USA). Multiple reaction monitoring (MRM) parameters for the ganciclovir, cimetidine, digoxin and ebesartan (internal standard, IS) were optimized and are summarized in Table S3. The other ionization parameters were as follows: curtain gas, 20 psi; collision gas, 6 psi; ion source gas 1, 50 psi; ion source gas 2, 50 psi, respectively, with a temperature of 500 °C and an ion spray needle voltage of 5500 V The bioanalytical method validation guidance for industry released by the FDA in 2018 was used to validate the analytical approach used in this study. The selectivity, specificity, accuracy, matrix effects, stability, served as key metrics to affirm the validity of this method [40].

### 4.8. Statistical Analyses

The experimental data were analyzed by Graphpad prism software (San Diego, CA, USA), and the mean value was calculated ± standard deviation (SD). The differences between groups (*p* < 0.05 and *p* < 0.01) were analyzed by SPSS 20.0. Dunnett multiple comparison test or LSD test were used for multiple comparison, *p* < 0.05 was regarded as statistically significant. The results of LC-MS/MS were analyzed by Das 2.0 software. Ccr was calculated with the following formula.

(1) $$Ccr=\frac{Urinary\;creatinine\times Urine\;volume\;mL}{Serum\;creatinine\;\left(\frac{mg}{mL}\right)\times \;Time\left(h\right)}$$

## 5. Conclusions

In conclusion, this experiment explored the relationship between major kidney transporters and creatinine, creatinine clearance, and renal fibrosis area. The development of modeling time in the TIF pathological model of rats was studied to infer the relationship between creatinine, creatinine clearance and kidney transporters. The results showed that OCT2 and MATE1 were negatively correlated with creatinine and fibrosis area, and positively correlated with creatinine clearance, while P-gp showed the opposite results. Therefore, we think that Cr/Ccr can be used to infer the transporter expression and renal fibrosis process. Using typical substrates for pharmacokinetic studies, the research results show that, with OCT2 lower expression, substrate cimetidine pharmacokinetic parameters show obvious changes in the body, with a notable rise in *AUC* and *Cmax*, while *Clr* was significantly down-regulated, suggesting that cimetidine excretion was significantly slowed in the TIF model. MATE1 and P-gp substrates showed the opposite results. Therefore, we believe that Cr/Ccr can be used as an indicator of OCT2, MATE1 and P-gp transporter expression, and its changes are significantly correlated with OCT2, MATE1 and P-gp changes, providing data and references for clinical renal disease patients in clinical medication.

## Figures and Tables

**Table 1:** Pharmacokinetic parameters of cimetidine, ganciclovir and digoxin.

Parameter	Cimetidine (*n* = 5)		Ganciclovir (*n* = 5)		Digoxin (*n* = 5)	
	Control	TIF	Control	TIF	Control	TIF
*AUC*_(0-t)_ mg/L·(min)	211,825.3 ± 25013.8	306,809.9 ± 61,720.3 *	3433.4 ± 665.0	3045.4 ± 583.9	1727.6 ± 775.3	417.5 ± 188.3 **
*AUC*_(0-∞)_ (mg/L·min)	215,600.9 ± 25,087.0	311,528.9 ± 59,201.0 *	3825.1 ± 669.6	3372.2 ± 687.3	2384.1 ± 1308.1	497.7 ± 183.2 *
*T*_max_ (min)	33.0 ± 6.7	33.0 ± 6.7	1.0 ± 0.1	0.8 ± 0.3	5.2 ± 3.9	2.8 ± 2.4
*C*_max_ (mg/L)	1133.1 ± 173.5	1982.1 ± 608.0 *	631.7 ± 196.2	1005.9 ± 297.1 *	99.0 ± 34.3	51.9 ± 29.3 *
*Cl*_r_ (mg/L·h)	0.05 ± 0.01	0.04 ± 0.01 **	0.012 ± 0.02	0.014 ± 0.003	0.003 ± 0.002	0.011 ± 0.005 **

TIF: The group of Model. Data are presented as mean ± S.E.M. Significance levels were evaluated using Student’s *t-*test or one-way ANOVA combined with Turkey’s multiple comparison test; ** *p* < 0.01, * *p* < 0.05.

## Data Availability

Not applicable.

## Supplementary Materials

Table S1: Methodology of Ganciclovir, Cimetidine and Digoxin, Table S2: The primer sequences of target genes and β-actin, Table S3: Optimized MRM parameters for analytes and IS.

## Sample Availability

Not available.

## References

[1] Zamek-Gliszczynski M.J., Patel M., Yang X., Lutz J.D., Chu X., Brouwer K.L.R., Lai Y., Lee C.A., Neuhoff S., Paine M.F. (2021). Intestinal P-gp and Putative Hepatic OATP1B Induction: International Transporter Consortium Perspective on Drug Development Implications. Clin. Pharmacol. Ther..

[2] Dalrymple L.S., Katz R., Kestenbaum B., Shlipak M.G., Sarnak M.J., Stehman-Breen C., Seliger S., Siscovick D., Newman A.B., Fried L. (2010). Chronic Kidney Disease and the Risk of End-Stage Renal Disease Versus Death. J. Gen. Intern. Med..

[3] Bönisch H. (2021). Substrates and Inhibitors of Organic Cation Transporters (OCTs) and Plasma Membrane Monoamine Transporter (PMAT) and Therapeutic Implications. Handb. Exp. Pharmacol..

[4] Sudsakorn S., Bahadduri P., Fretland J., Lu C. (2020). FDA Drug-Drug Interaction Guidance: A Comparison Analysis and Action Plan by Pharmaceutical Industrial Scientists. Curr. Drug Metab..

[5] Evans M., Lewis R.D., Morgan A.R., Whyte M.B., Hanif W., Bain S.C., Davies S., Dashora U., Yousef Z., Patel D.C. (2021). A Narrative Review of Chronic Kidney Disease in Clinical Practice: Current Challenges and Future Perspectives. Adv. Ther..

[6] Rana J.S., Khan S.S., Lloyd-Jones D.M., Sidney S. (2021). Changes in Mortality in Top 10 Causes of Death from 2011 to 2018. J. Gen. Intern. Med..

[7] Jia Y., Xu H., Yu Q., Tan L., Xiong Z. (2021). Identification and Verification of Vascular Cell Adhesion Protein 1 As an Immune-Related Hub Gene Associated with the Tubulointerstitial Injury in Diabetic Kidney Disease. Bioengineered.

[8] Wehrli F., Taneri P., Bano A., Bally L., Blekkenhorst L., Bussler W., Metzger B., Minder B., Glisic M., Muka T. (2021). Oat Intake and Risk of Type 2 Diabetes, Cardiovascular Disease and All-Cause Mortality: A Systematic Review and Meta-Analysis. Nutrients.

[9] Lee K.-H., Ou S.-M., Chu Y.-C., Lin Y.-P., Tsai M.-T., Tarng D.-C. (2021). Antithrombotic Therapy for Chronic Kidney Disease Patients with Concomitant Atrial Fibrillation and Coronary Artery Disease. Front. Cardiovasc. Med..

[10] McCarthy J.S., Yalkinoglu Ö., Odedra A., Webster R., Oeuvray C., Tappert A., Bezuidenhout D., Giddins M.J., Dhingra S.K., Fidock D.A. (2021). Safety, Pharmacokinetics, and Antimalarial Activity of the Novel Plasmodium Eukaryotic Translation Elongation Factor 2 Inhibitor M5717: A First-in-Human, Randomised, Placebo-Controlled, Double-Blind, Single Ascending Dose Study and Volunteer Infection Study. Lancet Infect. Dis..

[11] Barrera-Chimal J., Lima-Posada I., Bakris G.L., Jaisser F. (2021). Mineralocorticoid Receptor Antagonists in Diabetic Kidney Disease—Mechanistic and Therapeutic Effects. Nat. Rev. Nephrol..

[12] Smit C., Goulooze C., Brüggemann R.J.M., Sherwin C.M., Knibbe C.A.J. (2021). Dosing Recommendations for Vancomycin in Children and Adolescents with Varying Levels of Obesity and Renal Dysfunction: A Population Pharmacokinetic Study in 1892 Children Aged 1–18 Years. AAPS J..

[13] Wang S., Wang F., Wang X., Zhang Y., Song L. (2021). Elevated Creatinine Clearance in Lupus Nephritis Patients with Normal Creatinine. Int. J. Med. Sci..

[14] Schwalm S., Beyer S., Frey H., Haceni R., Grammatikos G., Thomas D., Geisslinger G., Schaefer L., Huwiler A., Pfeilschifter J. (2017). Sphingosine Kinase-2 Deficiency Ameliorates Kidney Fibrosis by Up-Regulating Smad7 in a Mouse Model of Unilateral Ureteral Obstruction. Am. J. Pathol..

[15] van der Made T.K., Fedecostante M., Scotcher D., Rostami-Hodjegan A., Toraño J.S., Middel I., Koster A., Gerritsen K.G., Jankowski V., Jankowski J. (2019). Quantitative Translation of Microfluidic Transporter in Vitro Data to in Vivo Reveals Impaired Albumin-Facilitated Indoxyl Sulfate Secretion in Chronic Kidney Disease. Mol. Pharm..

[16] Sirijariyawat K., Ontawong A., Palee S., Thummasorn S., Maneechote C., Boonphang O., Chatsudthipong V., Chattipakorn N., Srimaroeng C. (2019). Impaired Renal Organic Anion Transport 1 (SLC22A6) and Its Regulation Following Acute Myocardial Infarction and Reperfusion Injury in Rats. Biochim. Biophys. Acta Mol. Basis Dis..

[17] Takeda F., Oda M., Terasaki M., Ichimura Y., Kojima H., Saitoh H. (2021). Downregulated Expression of Intestinal P-Glycoprotein in Rats with Cisplatin-Induced Acute Kidney Injury Causes Amplification of Its Transport Capacity to Maintain “gatekeeper” Function. Toxicol. Appl. Pharmacol..

[18] Nishizawa K., Yoda N., Morokado F., Komori H., Nakanishi T., Tamai I. (2019). Changes of Drug Pharmacokinetics Mediated by Downregulation of Kidney Organic Cation Transporters Mate1 and Oct2 in a Rat Model of Hyperuricemia. PLoS ONE.

[19] Freitas-Lima L.C., Budu A., Arruda A.C., Perilhão M.S., Barrera-Chimal J., Araujo R.C., Estrela G.R. (2020). PPAR-α Deletion Attenuates Cisplatin Nephrotoxicity by Modulating Renal Organic Transporters MATE-1 and OCT-2. Int. J. Mol. Sci..

[20] Wang X., Deng J., Xiong C., Chen H., Zhou Q., Xia Y., Shao X., Zou H. (2020). Treatment With a PPAR-γ Agonist Protects Against Hyperuricemic Nephropathy in a Rat Model. Drug Des. Dev. Ther..

[21] Matheux A., Gassiot M., Fromont G., Leenhardt F., Boulahtouf A., Fabbrizio E., Marchive C., Garcin A., Agherbi H., Combès E. (2021). PXR Modulates the Prostate Cancer Cell Response to Afatinib by Regulating the Expression of the Monocarboxylate Transporter SLC16A1. Cancers.

[22] Sultana H., Kato A., Ohashi A., Takashima R., Katsurai T., Sato S., Monma M., Ohsaki Y., Goto T., Komai M. (2021). Effect of Vitamin K-Mediated PXR Activation on Drug-Metabolizing Gene Expression in Human Intestinal Carcinoma LS180 Cell Line. Nutrients.

[23] Li D., Cui Y., Wang X., Liu F., Li X. (2021). Apple Polyphenol Extract Improves High-Fat Diet-Induced Hepatic Steatosis by Regulating Bile Acid Synthesis and Gut Microbiota in C57BL/6 Male Mice. J. Agric. Food Chem..

[24] Liu F., Zhou J., Guo J., Huang W., Zhang W., Wang H. (2021). Prenatal Ethanol Exposure Increases Maternal Bile Acids through Placental Transport Pathway. Toxicology.

[25] El Kasmi K.C., Anderson A.L., Devereaux M.W., Balasubramaniyan N., Suchy F.J., Orlicky D.J., Shearn C.T., Sokol R.J. (2021). Interrupting Tumor Necrosis factor–alpha Signaling Prevents Parenteral nutrition–associated Cholestasis in Mice. J. Parenter. Enter. Nutr..

[26] Ambrus C., Bakos É., Sarkadi B., Özvegy-Laczka C., Telbisz Á. (2021). Interactions of Anti-COVID-19 Drug Candidates with Hepatic Transporters May Cause Liver Toxicity and Affect Pharmacokinetics. Sci. Rep..

[27] Elefantova K., Lakatos B., Kubickova J., Sulova Z., Breier A. (2018). Detection of the Mitochondrial Membrane Potential by the Cationic Dye JC-1 in L1210 Cells with Massive Overexpression of the Plasma Membrane ABCB1 Drug Transporter. Int. J. Mol. Sci..

[28] Yamazaki S., Costales C., Lazzaro S., Eatemadpour S., Kimoto E., Varma M.V. (2019). Physiologically-Based Pharmacokinetic Modeling Approach to Predict Rifampin-Mediated Intestinal P-Glycoprotein Induction. CPT Pharmacomet. Syst. Pharmacol..

[29] Qian C.-Q., Zhao K.-J., Chen Y., Liu X.-D. (2019). Simultaneously Predict Pharmacokinetic Interaction of Rifampicin with Oral Versus Intravenous Sub-Strates of Cytochrome P450 3A/P-Glycoprotein to Healthy Human Using a Semi-Physiologically Based Pharmacokinetic Model Involving Both Enzyme and Transporter Turnover. Eur. J. Pharm. Sci..

[30] Staples J.W., Stine J.M., Mäki-Lohiluoma E., Steed E., George K.M., Thompson C.M., Woodahl E.L. (2020). Food Dyes As P-Glycoprotein Modulators. Food Chem. Toxicol..

[31] Bailey D.G., Dresser G.K. (2004). Interactions Between Grapefruit Juice and Cardiovascular Drugs. Am. J. Cardiovasc. Drugs.

[32] Ito S., Kusuhara H., Yokochi M., Toyoshima J., Inoue K., Yuasa H., Sugiyama Y. (2011). Competitive Inhibition of the Luminal Efflux by Multidrug and Toxin Extrusions, But Not Basolateral Uptake by Organic Cation Transporter 2, Is the Likely Mechanism Underlying the Pharmacokinetic Drug-Drug Interactions Caused by Cimetidine in the Kidney. J. Pharmacol. Exp. Ther..

[33] Li S., Shu C., Wu S., Xu H., Wang Y. (2021). Population Pharmacokinetics and Dose Optimization of Ganciclovir in Critically Ill Children. Front. Pharmacol..

[34] Lal R., Sukbuntherng J., Luo W., Vicente V., Blumenthal R., Ho J., Cundy K.C. (2010). Clinical Pharmacokinetic Drug Interaction Studies of Gabapentin Enacarbil, a Novel Transported Prodrug of Gabapentin, With Naproxen and Cimetidine. Br. J. Clin. Pharmacol..

[35] Choi Y.A., Song I.-S., Choi M.-K. (2018). Pharmacokinetic Drug-Drug Interaction and Responsible Mechanism Between Memantine and Cimetidine. Pharmaceutics.

[36] Lai Y., Sampson K.E., Balogh L.M., Brayman T.G., Cox S.R., Adams W.J., Kumar V., Stevens J.C. (2010). Preclinical and Clinical Evidence for the Collaborative Transport and Renal Secretion of an Oxa-Zoli-Dinone Antibiotic by Organic Anion Transporter 3 (OAT3/SLC22A8) and Multidrug and Toxin Extrusion Protein 1 (MA-TE1/SLC47A1). J. Pharmacol. Exp. Ther..

[37] Jiménez-Uribe A.P., Bellido B., Aparicio-Trejo O.E., Tapia E., Sánchez-Lozada L.G., Hernández-Santos J.A., Fernández-Valverde F., Hernández-Cruz E.Y., Orozco-Ibarra M., Pedraza-Chaverria J. (2021). Temporal Characterization of Mitochondrial Impairment in the Unilateral Ureteral Obstruction Model in Rats. Free Radic. Biol. Med..

[38] You Y.-K., Wu W.-F., Huang X.-R., Li H.-D., Ren Y.-P., Zeng J.-C., Chen H., Lan H.Y. (2021). Deletion of Smad3 Protects Against Creactive Protein-Induced Renal Fibrosis and Inflammation in Obstructive Nephropathy. Int. J. Biol. Sci..

[39] Li A., Zhang X., Shu M., Wu M., Wang J., Zhang J., Wang R., Li P., Wang Y. (2017). Arctigenin Suppresses Renal Interstitial Fibrosis in a Rat Model of Obstructive Nephropathy. Phytomedicine.

[40] Chen J., Zhang X., Xie J., Xue M., Liu L., Yang Y., Qiu H. (2020). Overexpression of TGFβ1 in Murine Mesenchymal Stem Cells Improves Lung Inflammation by Impacting the Th17/Treg Balance in LPS-Induced ARDS Mice. Stem Cell Res. Ther..

