# Supplementary material for: The System Profile of Renal Drug Transporters in Tubulointerstitial Fibrosis Model and Consequent Effect on Pharmacokinetics
Source: Molecules. 2022 Jan 21;27(3):704. doi: 10.3390/molecules27030704 (PMC8838889; doi:10.3390/molecules27030704)
Supplement: Supplementary file 1 [file molecules-27-00704-s001.zip › molecules-1539101-supplementary.pdf]

Supplementary Materials:

Table S1 Methodology of Ganciclovir, Cimetidine and Digoxin

	Ganciclovir		Cimetidine		Digoxin	
	Concentration (ng/ml)		Concentration (ng/ml)		Concentration (ng/ml)	
Accuracy	50.0	9.43%	4.0	3.16%	50.0	5.12%
	500.0	6.2%	10.0	1.71%	500.0	4.65%
	2000.0	8.73%	200.0	1.65%	2000.0	7.21%
Stability	50.0	7.25%	4.0	6.13%	50.0	4.25%
	500.0	6.48%	10.0	6.81%	500.0	3.15%
	2000.0	8.21%	200.0	2.17%	2000.0	2.18%
Matrix effects	50.0	81.50%	3.12	70.60%	50.0	78.61%
	500.0	82.80%	50.0	70.80%	500.0	81.65%
	2000.0	88.60%	200.0	71.60%	2000.0	77.63%

Table S2 The primer sequences of target genes and *β-actin*

Name	Primer-Forward	Primer-Reverse	Reference
β-actin	TGACAGGATGCAGAAGGAGA	TAGAGCCACCAATCCACACA	(41)
Oat1	GTGGTTGCTCCCCTACTGCT	ATTCGGGTTGTCCTTGCTTG	Designed
Oat2	GCTGCATGATGGTGTGGTTT	CGGCGCACAAGGAAGTAGAC	(42)
Oat3	TCCTGGTGGGTACCAGAGTC	CTGCATTTCTGAAGGCACAA	Designed
Oatp4C1	GCAAGGTATTGTAGTAAATGGCCTAGT	AGACAACACGCAAAAGGAGATG	Designed
Oct1	TTCACCCCTGGACATTATTGC	TCATGCACTGGCTGAGGAAG	(43)
Oct2	CCCCAAACCCACACAAACC	CAACAGACCGTGCAAGCTACA	(42)
Mrp2	AAACGTTCACGGGCACATC	CAGGACTGCTGAGGGACATAGG	(44)
Mrp4	ATCCTCATACCCCTGGTTCC	TGCATCAAACAGCTCCTGAC	(42)
Bcrp	TCCAAGGTTGGAACTCAGTTTA	AAGATGGAATACCGAGGCTG	(45)
Mate1	TGCCATCGGCTATTATGTCAT	AGCTTGGCAACGAACATCAGT	Designed
Mdr1-α	GAGAGTGAAAAGGTCGTCCAGG	AAGTCTGCGTTCTGGATGGTG	(46)

Table S3 Optimized MRM parameters for analytes and IS

Analyte	Molecular formula	Precursor ion (m/z)	Product ion (m/z)	Declustering potential (V)	Collision energy(V)
Ganciclovir	$C_9H_{13}N_5O_4$	256.4	152.1	36	17
Cimetidine	$C_{10}H_{16}N_6S$	253.09	158.88	48	20
Digoxin	$C_{41}H_{64}O_{14}$	781.420	651.300	80	25
Ebesartan (IS)	$C_{25}H_{28}N_6O$	429.21	207.1	46	35
